# Advances in colorectal cancer screening: technological innovations, guideline discrepancies, and individualized strategies

**DOI:** 10.3389/fonc.2025.1723546

**Published:** 2025-11-28

**Authors:** Li Tang, Xiaoyong Zhao, Guohong Wang, Jiehao Huang, Mu Zhang, Wei Xu

**Affiliations:** 1 Department of Anesthesiology, the First Affiliated Hospital, Yangtze University, Jingzhou, Hubei, China; 2 Department of Ultrasound, the First Affiliated Hospital, Yangtze University, Jingzhou, Hubei, China

**Keywords:** colorectal cancer, Screening technology, Early detection, guideline discrepancies, Molecular diagnostics, artificial intelligence

## Abstract

Colorectal cancer (CRC) is the second leading cause of cancer-related mortality worldwide. Numerous clinical and epidemiological studies have demonstrated that early screening can significantly reduce both the incidence and mortality of CRC. This review systematically summarizes recent advances in CRC screening technologies. It first reviews the current applications of traditional screening tools such as colonoscopy and fecal occult blood tests, then focuses on emerging molecular detection techniques based on DNA, RNA, proteins, and metabolites, as well as representative multi-omics integration approaches. Furthermore, it discusses the innovative use of artificial intelligence (AI) and image recognition technologies in CRC screening. At the guideline level, we compare recent updates and implementation differences among major national screening guidelines, including those of the U.S. Preventive Services Task Force (USPSTF), and analyze key challenges in current screening practices. Finally, we propose directions for future development. By integrating existing evidence, this review aims to provide clinical reference for transforming CRC screening from population-based to precision-based individualized prevention, promoting its wide, efficient, and sustainable implementation.

## 1 Introduction

Colorectal cancer (CRC) is the third most common malignancy worldwide and the second leading cause of cancer-related death, posing a major public health burden. Epidemiological studies have revealed marked geographic and population-based variations in incidence and mortality, closely associated with genetic susceptibility, lifestyle, and dietary factors (1). In recent years, lifestyle changes have contributed to a continuous rise in CRC incidence, with an increasing trend toward younger onset (2). In China, CRC ranks first globally in both new cases and deaths, representing a particularly heavy disease burden (3).

Early screening is the cornerstone of CRC prevention and control, effectively reducing incidence and mortality. Standardized screening can increase early diagnostic rates by over 40% and markedly improve long-term prognosis (4). Current screening modalities include colonoscopy (the “gold standard”), fecal immunochemical test (FIT), and liquid biopsy based on circulating tumor DNA (ctDNA) (5–7). However, the efficacy and applicability of these methods vary among populations, and the adherence rate for colonoscopy remains below 50%, making optimization of screening strategies essential (8, 9).

With the advancement of artificial intelligence (AI) and genomics, CRC screening is undergoing continuous innovation (10, 11). AI-assisted colonoscopy has been shown to significantly improve adenoma detection rates (ADR). A meta-analysis including 6,800 cases reported an odds ratio of 1.51 in favor of AI-assisted detection (12). These technologies provide new avenues for developing individualized screening strategies and improving early detection efficiency (13). This review systematically summarizes recent advances in CRC screening technologies, integrating epidemiological and public health perspectives to explore future research directions and technological innovations.

## 2 Traditional screening methods

### 2.1 Colonoscopy and sigmoidoscopy

Colonoscopy remains the gold standard for CRC screening, allowing simultaneous detection and removal of polyps, with high diagnostic accuracy (14). However, its complexity, time requirement, and operator dependence limit widespread use (15). Sigmoidoscopy is easier, better tolerated, and requires minimal preparation but only assesses the distal colon, risking missed right-sided lesions (16). These methods are complementary: colonoscopy suits high-risk individuals, while sigmoidoscopy can serve as a primary community screening tool. **Table 1** summarizes key features of common CRC screening modalities.

**Table 1:** Comparison of several common colorectal screening methods.

Dimension	Colonoscopy	Sigmoidoscopy	FOBT/FIT	Multi-target stool DNA test
Screening value for CRC	Gold standard: enables full visualization of the colon, detection and removal of polyps, significantly reducing CRC incidence	Moderate: covers only distal colon; may miss proximal lesions	Non-invasive initial screening method; FIT shows higher sensitivity than gFOBT for early cancer and advanced adenomas	High sensitivity for CRC (93.9%) and advanced precancerous lesions (43.4%); higher than FIT but slightly lower specificity
Diagnostic & therapeutic purpose	Both diagnostic and therapeutic; allows biopsy and polyp removal during the same procedure	Mainly used for distal colorectal lesion evaluation; suitable for preliminary or complementary screening	Detects occult bleeding; suitable for population-based screening	Combines methylated DNA and hemoglobin detection to improve early diagnostic accuracy
Advantages	High diagnostic accuracy; allows immediate intervention	Simple, minimal preparation, and well tolerated	Non-invasive, low cost, easy sampling, widely accepted	High sensitivity; integrates molecular and immunologic biomarkers; quantitative and automated
Limitations	Invasive; requires skilled operators; limited scalability in large populations	Limited detection range; cannot assess proximal colon	gFOBT has low sensitivity and is affected by diet or medication; repeated testing needed	Slightly lower specificity; high cost and complex laboratory workflow
Clinical recommendation	Recommended for high-risk or symptomatic individuals	Suitable for preliminary screening in primary care or low-resource settings	FIT is recommended by international guidelines as the first-line non-invasive CRC screening method	Included in several national guidelines as an alternative to FIT

### 2.2 Fecal occult blood test and fecal immunochemical test

FOBT was the earliest non-invasive CRC screening method, including the guaiac-based test (gFOBT) and the immunochemical test (FIT). The gFOBT detects the peroxidase activity of hemoglobin via a guaiac reaction but has low sensitivity and is affected by diet and medications (17–19). FIT employs antibodies against human hemoglobin, offering higher specificity and minimal interference from external factors (20). Studies have shown that FIT significantly outperforms gFOBT in detecting early CRC and advanced adenomas. Adjusting the cut-off value allows for individualized balancing between sensitivity and specificity (21, 22). Owing to its superior accuracy and convenience, FIT has been endorsed by international guidelines as the preferred fecal-based CRC screening method (23).

## 3 DNA-based and biomarker detection

### 3.1 DNA methylation testing

With the progress of molecular diagnostics, DNA methylation testing has shown great promise in CRC screening. This technique identifies methylation signatures in tumor-derived DNA from stool or blood samples, enabling the detection of early cancer and precancerous lesions (24, 25). Common biomarkers include mSEPT9, mNDRG4, and SFRP2, all of which demonstrate strong diagnostic performance (26, 27).

mSEPT9, one of the most extensively validated markers, captures cancer signals through methylation of the SEPT9 promoter region in ctDNA (28). It exhibits high sensitivity for detecting CRC and advanced adenomas, particularly useful for individuals unable or unwilling to undergo colonoscopy (29). Methylated mNDRG4 is strongly associated with CRC development; compared with FIT, it significantly improves early cancer and advanced adenoma detection rates while reducing false positives (30, 31). SFRP2, a regulator in the Wnt signaling pathway, is frequently silenced by promoter methylation, contributing to tumorigenesis (32–34). Combined detection of multiple methylation markers yields synergistic effects, substantially improving both sensitivity and specificity (35, 36). **Table 2** summarizes biomarker-based colorectal cancer screening methods.

**Table 2:** Summary of biomarker-based colorectal cancer screening methods.

Biomarker type	Detection technique	Advantages	Limitations	Representative studies/products	Overall evaluation
Stool DNA Methylation (mNDRG4, SFRP2)	Methylation PCR, NGS	Clear tissue origin; high cancer specificity; sensitive for early detection	Weak signal in some cancers(sarcoma); sample quality affected by stool preservation	Cologuard, Coloclear	Widely studied and clinically applied; methylation-based NGS is a core technology in MCED
Blood DNA Methylation (mSEPT9, SDC2)	Methylation-specific PCR (MSP), bisulfite sequencing, targeted NGS	Minimally invasive; suitable for early detection; high CRC-specific methylation signature specificity.	Low cfDNA concentration in early-stage CRC; possible interference from non-tumor cfDNA; requires high analytical sensitivity	ColonAiQ, Shield, Epi proColon, ColoVantage	Clinically validated and increasingly adopted; promising for population-level CRC screening and MRD monitoring
ctDNA Mutation	dPCR, NGS	Directly reflects driver gene alterations; valuable for targeted therapy guidance	Low abundance in early stages; affected by clonal hematopoiesis; difficult to trace origin	ColoScape, CancerGuard	Requires combination with other biomarkers to improve sensitivity; limited value when used alone
Circulating Tumor DNA (ctDNA)	dPCR, NGS	Enables dynamic monitoring of recurrence risk; noninvasive evaluation of treatment response; prognostic prediction	Low ctDNA levels in early tumors; high testing cost	Guardant360	Expanding clinical applications; combination with CEA improves recurrence risk stratification
Multitarget stool-RNA (mt-sRNA)	qRT-PCR or ddPCR	Reflects gene-expression changes in exfoliated intestinal cells; suitable for early CRC screening	RNA instability; high sample processing requirements; limited sensitivity for advanced adenomas	ColoSense	First RNA-based noninvasive CRC screening test FDA-approved (PMA P230001, 2024); clinically promising but requires further real-world validation
Circulating cell-free RNA (cfRNA)	RNA-seq, qRT-PCR	Higher sensitivity than cfDNA; extracellular vesicle RNA is more stable	Risk of sample degradation; complex analytical procedures	ThromboSeq(TEP RNA, blood-based liquid biopsy)	Emerging liquid biopsy approach; strong potential for multi-omics integration
Protein Biomarkers(CEA, CRP, GZMB, MMP12)	Mass spectrometry, Olink platform	Mature technology; rapid detection;several proteins show clinical relevance	Limited sensitivity and specificity; single protein cannot reflect overall changes	OncoSeek	Traditional and novel combined strategies in use; facilitates multi-omics diagnostic approaches
Metabolites & Microbiota-Derived Metabolites	Mass spectrometry, 16S rRNA sequencing	Reflects metabolic state; reveals CRC-related microbiota and immune pathways	Complex metabolic flux; easily affected by diet	MNALCI	High interpretability; promising for CRC progression monitoring and immune response evaluation
Multi-omics Integration	Combined NGS, proteomics, metabolomics and AI analytics	Enhanced overall performance; complementary signal coverage across omics layers	High cost; complex data processing; difficult biological interpretation	AlphaLiquid, Freenome	Offers comprehensive detection and systemic insights; cost-benefit balance required; suitable as a complementary approach

### 3.2 Next-generation multi-target fecal DNA testing

Next-generation multi-target fecal DNA testing integrates methylation biomarkers with hemoglobin detection (37). In a prospective study involving 20,176 asymptomatic individuals aged ≥40 years, the test achieved a sensitivity of 93.9% for CRC, 43.4% for advanced precancerous lesions, and a specificity of 90.6% for advanced neoplasia. Compared with FIT, it demonstrated significantly higher sensitivity for CRC and advanced precancerous lesions (*P*<0.001), with a slight decrease in specificity (*P*<0.001) but no major adverse events (38). This method has already been included in screening guidelines in several countries.

### 3.3 Circulating tumor DNA testing

Cell-free DNA (cfDNA) refers to double-stranded DNA fragments released from apoptotic or necrotic cells into the bloodstream. Tumor cells also release cfDNA carrying tumor-specific genetic information, known as ctDNA (39). As an emerging liquid biopsy approach, ctDNA testing can identify tumor-related genetic alterations in blood, making it suitable for recurrence monitoring and therapeutic response evaluation (40, 41).

Mo S et al. (25) analyzed six methylation markers in ctDNA from pre- and postoperative samples of 299 patients with stage I–III CRC. Preoperatively, 78.4% of patients were ctDNA-positive; one month post-surgery, ctDNA positivity was associated with a 17.5-fold higher risk of recurrence. Combining ctDNA with CEA further optimized risk stratification. Dynamic postoperative monitoring revealed that ctDNA-positive patients relapsed earlier, with ctDNA detection preceding radiologic evidence by approximately 3.3 months, suggesting strong potential for early recurrence prediction and individualized management.

## 4 Detection of RNA and its biomarkers

### 4.1 miRNA and gut microbiota detection

MicroRNAs (miRNAs) are a class of non-coding RNAs that regulate gene expression by binding to target mRNAs, thereby influencing cell proliferation, differentiation, and apoptosis. Studies have shown that miRNAs play critical roles in CRC initiation, progression, metastasis, and treatment response (42, 43). Analysis of fecal miRNA expression profiles indicates that specific miRNA signatures are closely associated with CRC occurrence and can serve as potential biomarkers for early screening and prognosis evaluation (44).

Meanwhile, gut microbiota profiling based on 16S rRNA sequencing has revealed distinct microbial signatures in CRC patients (45), which show potential for clinical staging and KRAS mutation prediction. Liu J et al. (46) analyzed preoperative fecal samples from 192 CRC patients and found that Simpson diversity indices were lower in stage III–IV cases, with enrichment of Proteobacteria. O-glycan biosynthesis pathways were strongly associated with tumor progression. Microbiota-based models effectively distinguished cancer stages, suggesting that specific bacterial taxa may contribute to tumor progression through immune modulation or endoplasmic reticulum stress mechanisms.

### 4.2 Circulating cell-free RNA detection

Circulating cell-free RNA (cfRNA) is an emerging non-invasive biomarker for CRC detection. Plasma cfRNA sequencing identifies CRC-specific transcriptomic signatures, achieving high sensitivity and specificity in distinguishing early-stage CRC from healthy individuals (47). Analysis of microbe-derived cfRNA modifications further improves discrimination between CRC and non-CRC samples (48). Compared with cfDNA, cfRNA provides higher detection sensitivity and richer pathway information, highlighting its advantages in multi-omics blood-based surveillance (49, 50).

Advancements in low-input methylation sequencing and optimized library construction enhance cfRNA detection efficiency and coverage (51, 52). Using DETECTOR-seq, Wang H et al. systematically analyzed cfRNA profiles from plasma and extracellular vesicles (EVs) (53). Plasma was enriched in circular RNAs, tRNAs, Y RNAs, and viral RNAs, whereas EVs contained more mRNAs and srpRNAs. Both sources effectively distinguished cancer patients from healthy controls, and microbial RNA signatures in plasma showed strong potential for cancer-type classification.

## 5 Protein and metabolite biomarkers

### 5.1 Blood protein biomarkers

Blood protein biomarkers, such as carcinoembryonic antigen (CEA) and C-reactive protein (CRP), have been widely employed in colorectal cancer (CRC) research. Using Olink proteomics technology, researchers have identified numerous protein biomarkers with potential diagnostic value. For instance, in saliva samples, the expression levels of GZMB (granzyme B) and MMP12 (matrix metalloproteinase 12) are significantly altered in CRC patients, demonstrating high diagnostic sensitivity and specificity (54). Furthermore, serum proteomics analyses have revealed abnormal expression of coagulation factor XIII A chain (F13A1) and plasma kallikrein (KLKB1) in CRC patients, which are closely associated with tumour progression (55). Studies have also reported that plasma concentrations of several amino acids (including L-valine, L-threonine, L-methionine, and glycine) are abnormal in CRC patients, reflecting dysregulated energy metabolism and tumour cell proliferation (56, 57).

### 5.2 Microbiota-derived metabolites

Extensive evidence indicates that the gut microbiota and their metabolites play key regulatory roles in CRC initiation and progression. These metabolites, derived from microbial catabolism of dietary nutrients and host components, can influence tumorigenesis and microenvironment remodeling through multiple pathways. Short-chain fatty acids, bile acids, tryptophan metabolites, and polyamines produced by gut microbes can promote CRC by modulating inflammation, immune responses, and cellular metabolism (58, 59). Emerging metabolites, such as hydrogen sulfide (H_2_S) and formate, have recently drawn attention for their roles in CRC pathogenesis (60, 61). Yue T et al. (62) demonstrated that H_2_S is a critical factor affecting CRC immunotherapy efficacy; high expression in tumour tissue interferes with immune balance via protein persulfidation, promoting Treg activation and inhibiting CD8^+^ T-cell migration. Reduction of H_2_S levels can reverse these effects, improving the tumour immune microenvironment and enhancing checkpoint inhibitor efficacy. Ternes D et al. (63) further confirmed that formate produced by nucleated Clostridia can reprogramme CRC cell metabolism, activate pro-invasion signaling pathways, and enhance tumour invasiveness and inflammatory infiltration, accelerating CRC progression.

## 6 Several representative screening techniques and detection platforms

### 6.1 CancerGuard technology

CancerGuard (previously known as CancerSEEK) is a representative multi-cancer early detection (MCED) approach, integrating liquid biopsy analysis of circulating tumour DNA (ctDNA) mutations with plasma protein biomarkers to achieve multi-cancer screening, including CRC (64). CancerGuard uses a multi-analyte approach, combining genomic alterations and protein biomarker quantification. By analyzing around 2000 unique genomic loci, it identifies mutations, deletions, amplifications, and other alterations specific to cancer cells. Alongside this, it quantifies eight key protein biomarkers linked to cancer progression and metastasis. The integration of genomic and proteomic data provides a comprehensive view of the tumor’s molecular landscape. This approach helps in early cancer detection, personalized treatment strategies, and monitoring therapy response or recurrence. The assay was originally developed by the Johns Hopkins University team and was rebranded as CancerGuard following its commercial advancement in recent years to reflect updated analytical and clinical validation progress. Cohen JD et al. (65) applied **CancerGuard** to 1,005 early-stage cancer patients across eight tumour types, reporting an overall median positive rate of 70%. Sensitivity for five cancers lacking routine screening ranged from 69% to 98%, specificity in healthy controls exceeded 99%, and 83% of positive cases could be localised to the potential tumour site. These findings confirm **CancerGuard** potential for non-invasive CRC screening, although independent validation remains necessary.

### 6.2 DELFI method

DELFI (DNA Evaluation of Fragments for Early Interception) is a liquid biopsy technology based on cell-free DNA (cfDNA) fragmentomics. It employs low-pass whole-genome sequencing (WGS) to comprehensively analyse cfDNA fragment features, including size distribution, end motifs, and nucleosome positioning, combined with machine learning algorithms to identify tumour-associated DNA fragment abnormalities. Its advantage lies in tumour detection without reliance on specific gene mutation analysis (66).

Studies show that DELFI-TF (Tumour Fraction) scores are highly correlated with ctDNA levels (r=0.90), effectively estimating tumour burden even when mutations are undetected, enabling monitoring of tumour dynamics (67).

### 6.3 Cologuard

Cologuard is primarily designed for colorectal cancer (CRC) screening, analyzing stool samples for human hemoglobin and methylated DNA biomarkers, while its reliance on early mutation-based targets has been reduced in the current version (68). The updated multitarget stool DNA test (Cologuard Plus) demonstrated a sensitivity of 93.9% (95% CI, 87.1–97.7) for CRC and 43.4% (95% CI, 41.3–45.6) for advanced precancerous lesions, with a specificity of 90.6% (95% CI, 90.1–91.0) in asymptomatic adults (69).

### 6.4 Shield test

The **Shield Test** is a blood-based colorectal cancer (CRC) screening assay developed by Guardant Health, employing cell-free DNA (cfDNA) methylation analysis combined with machine learning for non-invasive detection of CRC and advanced precancerous lesions (70). In a large prospective study (10,258 participants), the Shield test achieved **sensitivity of 83% for CRC** and **specificity of 90%** for advanced neoplasia detection, demonstrating clinical performance comparable to FIT but with greater patient compliance due to its blood-based nature. It represents an emerging non-invasive alternative to stool-based tests such as Cologuard and ColoSense, marking a significant step toward precision CRC screening through epigenomic profiling (71).

### 6.5 Multi-cancer early detection platform

Galleri is a blood-based **multi-cancer early detection (MCED) platform** that analyses cfDNA methylation patterns using machine learning to detect signals from multiple cancer types and predict tissue of origin (72, 73). It can detect over 50 tumour types with specificity exceeding 99%, a false-positive rate below 1%, overall sensitivity of 54.9%, early-stage (I–III) sensitivity of 43.9%, and 93% accuracy for tissue-of-origin prediction (74). As an MCED platform, Galleri is distinct from CRC-specific assays, such as Cologuard or ColoSense, which focus solely on colorectal cancer and advanced precancerous lesions. Galleri embodies a “breadth-first” strategy for multi-cancer detection, complementary to the “depth-first” approach of CRC-targeted assays.

### 6.6 Freenome multi-omics platform

Freenome is a blood-based multi-omics screening platform that integrates cell-free DNA (cfDNA) methylation, fragmentomics features, and plasma proteomics data, combined with machine learning algorithms to identify early cancer signals.

In a recent large-scale study, the Freenome colorectal cancer (CRC) screening test demonstrated a **sensitivity of 79.2%** for CRC detection and a **specificity of 91.5%** for advanced tumors. The **negative predictive value (NPV)** for advanced CRC was **90.8%**, and the **positive predictive value (PPV)** was **15.5% (**75). These findings indicate that multi-omics integration models outperform single-omics approaches in early cancer detection.

## 7 Artificial intelligence and imaging recognition in CRC screening

### 7.1 AI-Assisted colonoscopy systems

AI-based imaging recognition has been widely applied in CRC screening, particularly in colonoscopy, significantly reducing missed lesions. Multicentre RCTs have demonstrated that AI-assisted colonoscopy systems (e.g., the “Eagle-Eye” system) can increase adenoma detection rates (ADR) from 32.4% to 39.9%, and improve detection of advanced adenomas (76). A multicentre trial in China also showed that CADe systems significantly increased the number of adenomas detected per procedure (APC) and polyp detection rate (PDR) (77). A 2024 RCT using RetinaNet-based AI further confirmed superior PDR and ADR outcomes compared with conventional methods (78). Beyond detection, AI shows potential in polyp characterisation and optical biopsy, enabling differentiation of benign and malignant lesions and prediction of invasion (79).

### 7.2 AI-Assisted computed tomography colonography and 3D reconstruction

CT colonography (CTC) is a non-invasive low-dose spiral CT method that provides “virtual endoscopy” through 2D and 3D reconstructions, enabling detection of polyps, strictures, and tumours (80). Studies indicate that CTC sensitivity for ≥10 mm adenomas or cancers is comparable to conventional colonoscopy, with detection of 6–9 mm lesions continuously improving (81). Modern Deep-Learning Reconstruction (DLR) technology significantly enhances image signal-to-noise ratio and reduces artefacts at low radiation doses, providing high-quality inputs for 3D surface reconstruction, thereby improving lesion visualisation and detection accuracy (82). Importantly, as a non-invasive and sedation-free technique, CTC reduces the need for anesthesia-assisted procedures, improving patient comfort and compliance while maintaining diagnostic accuracy.

### 7.3 Deep learning and multimodal data integration

AI and multimodal data fusion have demonstrated notable potential in CRC screening and precision treatment. Deep learning models can extract complex imaging features to predict prognosis and treatment response. Multi-stain deep learning models (MSDLM) analysing tumour immune microenvironments outperform traditional indices in survival prediction (83); models based on H&E images can identify consensus molecular subtypes (CMS), informing personalised therapy (84). Multimodal models integrating MRI, pathology, and clinical data offer comprehensive disease characterisation, with multicentre studies showing improved survival prediction accuracy (C-index=0.86) (85). Multi-task deep learning excels in tumour segmentation and treatment response prediction, with pCR identification AUC reaching 0.95 (86). Weakly supervised models combining MRI and pathology have achieved lymph node diagnostic accuracy approaching expert levels (87).

## 8 Key updates and implementation differences in international screening guidelines

The latest USPSTF update highlights the increasing incidence of early-onset colorectal cancer (EOCRC) and recommends lowering the screening initiation age from 50 to 45 years, while maintaining FIT and colonoscopy as core modalities. In contrast, several European countries (e.g., Germany) continue to start at 50 years, emphasizing early intervention in genetically high-risk groups (88). Implementation varies globally due to differences in cost-effectiveness, coverage, and healthcare resources. In low-resource regions, FIT is preferred for its affordability and non-invasiveness, though colonoscopy adherence remains low (30%–60%). Despite public awareness efforts, screening coverage in some areas remains below 40%, reflecting inequitable resource allocation (89).

## 9 Current challenges in colorectal cancer screening

Despite proven mortality reduction, global CRC screening participation remains suboptimal, influenced by inadequate public awareness, lack of physician recommendation, and fear of colonoscopy. Novel biomarkers such as circulating tumour DNA (ctDNA) exhibit high sensitivity but limited capacity for detecting precancerous lesions; for example, the PREEMPT CRC study reported a sensitivity of only 12.5%, indicating the need for further clinical validation (90). Additionally, AI applications in screening face challenges related to algorithm interpretability, data privacy and security, and population generalisability, raising ethical and regulatory concerns (91). Improving public adherence, optimising biomarker technologies, and establishing robust AI regulatory frameworks are pivotal to advancing CRC screening.

## 10 Limitations

This review has several limitations. Rapid technological advances mean some recent data may be excluded. Study heterogeneity also restricts direct comparison across platforms. Moreover, no quantitative meta-analysis was performed. Future research should update datasets and use standardized evaluation methods to enhance comparability.

## 11 Summary and perspectives

CRC screening is moving towards personalized, precision strategies integrating genetics, lifestyle, and environment. In parallel, the principles of individualized management in anesthesiology offer valuable insights for personalized CRC screening and perioperative strategies. In anesthetic practice, patient-specific physiological variability, genetic differences in drug metabolism, and comorbidity profiles are increasingly integrated into precision anesthesia protocols. Similarly, individualized CRC screening should account for genetic susceptibility, metabolic status, and systemic inflammatory responses that may influence both cancer risk and perioperative outcomes. The convergence of precision anesthesiology and precision oncology underscores a broader trend toward data-driven, individualized medicine, highlighting the importance of integrating multidisciplinary insights into CRC prevention and management.

Early genetic testing (e.g., APC, MLH1) in high-risk groups improves early detection (92). Multi-omics approaches combining plasma proteomics, ctDNA, and machine learning enhance early lesion prediction (93, 94). AI-driven multimodal integration further supports dynamic, individualized management, while real-time imaging with deep learning optimizes detection and treatment response (95). Future research should prioritize validating emerging multi-omics assays, developing interpretable and generalizable AI models, and exploring the integration of CRC screening with perioperative and anesthetic management to advance personalized cancer care.
